# Irritable bowel syndrome in Egyptian medical students, prevalence and associated factors: a cross-sectional study

**DOI:** 10.11604/pamj.2022.41.311.28228

**Published:** 2022-04-18

**Authors:** Shimaa Mahmoud El Sharawy, Ibrahim Fathi Amer, Mahmoud Zaki Elkadeem

**Affiliations:** 1 Department of Tropical Medicine and Infectious Diseases, Faculty of Medicine, Tanta University, Tanta, Egypt,; 2 Department of Hepatology, Gastroenterology and Infectious Diseases, Faculty of Medicine, Kafr El Sheikh University, Kafr El Sheikh, Egypt

**Keywords:** Irritable bowel syndrome, Rome III criteria, medical students, depression

## Abstract

**Introduction:**

irritable bowel syndrome is a recurrent chronic gastrointestinal functional disorder. Despite it is not dangerous; it carries a significant feedback on self-confidence and quality of life. Medical students are expected to develop irritable bowel syndrome because they are subjected to stress due to over academic pressure. The objectives were to investigate irritable bowel syndrome prevalence, and to detect the related risk factors in this specific group of Egyptian people.

**Methods:**

this cross-sectional study performed in two faculties of medicine in Nile Delta, Egypt. It had been built on self-administered questionnaires including Rome III criteria for diagnosis of irritable bowel syndrome, as well as several questions for gathering socio-demographic information and manifestations suggesting irritable bowel syndrome.

**Results:**

fifty (27.5%) of 182 evaluated medical students achieved criteria of irritable bowel syndrome, 64% of them were mixed type. Irritable bowel syndrome had a significant relationship with coffee, milk products, fewer vegetables, and fruits intake (P=0.034, P=0.044, P<0.001 respectively). Depression, anxiety, and food intolerance were detected to be significantly related to irritable bowel syndrome (p<0.001, p=0.005, p=0.04) respectively.

**Conclusion:**

it was demonstrated that many Egyptian medical students were suffering from irritable bowel syndrome. Some dietary habits, anxiety, and depression of the students could be risk factors related to development of irritable bowel syndrome.

## Introduction

Irritable bowel syndrome (IBS) is described as recurrent chronic gastrointestinal functional disturbances. IBS patients are often presented with abdominal discomfort, distension, and alteration in bowel behaviour in the form of constipation, diarrhoea, or both predominant [1, 2]. At present, the Rome III criteria are the main method for IBS diagnosis without the need for any biochemical markers, provided that warning signs have to be excluded [3, 4]. While IBS is not actually a life-threatening problem, it has been recorded that its symptoms significantly undermine the quality of life associated with health including psychological status and manifestation of fears [5, 6].

IBS affects approximately (10-25) percent of the global population [2]. Besides this, its symptoms are among the most common causes of primary health care consultations [7]. Nevertheless, in the United States alone, 2.4-3.5 million persons visit doctors yearly because of IBS [8]. Recently, prevalence of IBS has been reported to 9.2 percent in fifty-three studies made in 38 countries and included 395,385 participants [9]. In western nation's general population, prevalence of IBS was estimated to be between 10 to 18% [10, 11]. Whereas in non-western countries, IBS has earned less attention. The prevalence of IBS reaches 35-43% in some developing countries [12, 13].

IBS seems to be more likely to affect younger individuals overall [14]. Many university students; notably those studying medicine, may be affected by depression and anxiety [15]. Medical students are suffering from many factors including high levels of psychological stress due to large concentrated study, examination load, increased fierce work competitive environment, uncomfortable living, dietary practices, and patient management responsibilities [16]. There is a little knowledge about the spread of IBS in Egypt as well as other Arabian countries, specifically among university students. In addition, there are really limited data about the prevalence and contributing factors of IBS in university students, particularly in the medical field [17]. So the study aimed to seek for the prevalence of IBS and to identify risk factors associated with it among medical students in two faculties of medicine in both Tanta and Kafr El-Sheikh Universities.

## Methods

**Study design, setting and participants:** this was a cross-sectional study carried out in January and February 2021. The study included 182 medical students whether under or postgraduate and aged 18 years or more in Tanta and Kafr El-Sheikh Universities, Egypt, in the academic year (2020/2021). All participants signed an informed consent form prior to participation, after explanation of the study. The Ethical Review Board of Kafr El-Sheikh University has approved the study (Approval code: MKSU 9-12-20). The study protocol was in line with the ethical guidelines of the 1975 Declaration of Helsinki. Students with known organic gastrointestinal diseases or complaining of alarming gastrointestinal symptoms as weight loss or bloody stools, or individuals who did not desire to participate in the study, were excluded. The sample size was calculated by Raosoft Sample Size Calculator for this study according to 95% confidence interval, hypothesized percentage of IBS in the population =13%, and margin of error 5% as 174.

**Study variables and data sources:** some variables were evaluated through a confidential, self-administered, and reliable, questionnaire consisted of historical data and sociodemographic data (e.g. age, gender, academic year, special habits, hours of sleeping, food habits, food intolerance, family history, number of siblings, and birth order). In addition, participants were asked if they complained of some symptoms (e.g. abdominal pain, heartburn, vomiting, bowel habits, urinary symptoms), and asked about history of chronic diseases, and medications they were used to taking.

**Data collection and processing:** the questionnaires were in English language to maintain clarity and uniformity. Ten students were pre-tested before collection of data, and the data of pre-test were excluded from final analysis. The participants were approached by going from class-to-class. Irritable bowel syndrome (IBS) was diagnosed by application of English version of Rome III criteria [18]: recurrent abdominal pain or discomfort remaining three days or more monthly and has been reported in the last three months plus two or more of these criteria: (a) associated with altered frequency of stool, (b) altered stool appearance or consistency with the onset of the condition, and (c) the condition relieved after defecation. Symptoms suggesting IBS included altered frequency of stool, urgency, straining, abdominal bloating, and change in stool consistency. The participants of the study were grouped as one group had IBS and another group without IBS.

**Statistical methods:** statistical analysis was done using SPSS (version 22.0, IBM, New York, USA). Categorical data were analysed using the Chi-square test with Yate's (continuity) correction. Fisher's exact test was used if one or more expected value <5. Multivariate analysis using binary logistic regression was done to evaluate the significance of risk factors for associated with irritable bowel syndrome. The results of the regression analysis were reported using adjusted odds ratio and a 95% confidence interval.

## Results

**Criteria of participants:** this research was conducted on 182 medical students. They were between 21-30 years old, with a mean age (22.87+1.64). Males were 76 (41.8%), and females were 106 (58.2%). There were 62 (34.1%) from rural areas and 120 (65.9%) from urban areas. One hundred seventy (93.4%) were single.

**Prevalence of irritable bowel syndrome:** according to Rome III criteria, irritable bowel syndrome (IBS) was diagnosed in 50 (27.5%) of the study sample. As regards the IBS group, 8 (16%) students had constipation dominant IBS, 10 (20%) had diarrhoea dominant IBS, and 32 (64%) had mixed IBS. No significant statistical difference between the IBS patients and non-IBS ones as regard to age, gender, residence and family history (Table 1).

**Table 1:** baseline demographic criteria of the studied groups

Criteria	Total	Irritable bowel syndrome		P – value
	(n=182)	No (n=132)	Yes (n=50)	
Age (years) mean + SD	22.87+1.64	22.95+1.71	22.64+1.43	0.248
**Gender**				0.140
Female	106	72	34	
Male	76	60	16	
**Residence**				0.870
Rural	62	44	18	
Urban	120	88	32	
**Smoking**				#1.000
Yes	8	6	2	
No	174	126	48	
**Marital status**				#0.739
Single	170	124	46	
Married	12	8	4	
**Sleeping**				0.088
< 8 hours	86	68	18	
> 8 hours	96	64	32	
**GPA**				#0.175
Accepted	2	2	0	
Good	22	16	6	
Very good	40	34	6	
Excellent	118	80	38	
**Family history IBS**				0.72
Yes	60	42	18	
No	122	90	32	
**Number of Sibling**				#0.012*
0	8	6	2	
1	4	4	0	
2	80	52	28	
3	66	56	10	
4	18	12	6	
5	4	2	2	
9	2	0	2	

* Significant # Fisher's Exact Test; Other P values for categorical data were calculated by Chi-square test with Yates correction. Quantitative data were calculated by Student t test

**Associates of irritable bowel syndrome:** as demonstrated in (Table 2), no significant association was revealed between IBS and age, sex, marriage, smoking status, family status, and academic degree. From (Table 3), it could be detected that IBS was significantly associated with some feeding habits (number of daily meals (P =0.011), food intolerance (P =0.04), caffeine intake (P =0.034), milk products' intake (P =0.044), and vegetables and fruits intake (P< 0.001)). However, habits like drinking within meals, fried and seafood intake and chocolate intake were not significantly associated with IBS.

**Table 2:** feeding habits of the studied groups

Criteria	Total	Irritable bowel syndrome		P – value
	(n=182)	No (n=132)	Yes (n=50)	
**Number of meals**				#0.011*
2	34	18	16	
3	104	82	22	
4	36	28	8	
5	8	4	4	
Drinking within meals	112	76	36	0.106
Food intolerance	35	20	15	0.040*
Caffiene intake	114	76	38	0.034*
Fatty or fried food	112	78	34	0.351
Milk products	100	66	34	0.044*
Spicy food	80	54	26	0.239
Chocolates	86	62	24	1.000
Sea food	28	20	8	1.000
Vegetables and fruits	132	108	24	<0.001*

* Significant #Fisher's Exact Test; Other P values for categorical data were calculated by Chi-square test with Yates correction.

**Table 3:** clinical data of the studied groups

Criteria	Total	Irritable bowel syndrome		P – value
	(n=182)	No (n=132)	Yes (n=50)	
BMI				#0.792
Underweight	12	10	2	
Average	112	82	30	
Overweight	46	32	14	
Obese	12	8	4	
Weight loss	54	32	22	0.015*
Epigastric pain	62	30	32	<0.001*
Early satiety	70	44	26	0.032*
Heart burn	44	24	20	0.004*
Vomiting	24	8	16	<0.001*
Urinary symptoms	30	22	8	1.000
Hyperthyroidism	4	4	0	#0.580
Hypertension	4	2	2	#0.3
Anxiety	22	8	14	#<0.001*
Depression	8	2	6	#0.005*
Anaemia	22	16	6	1.000
Medical therapy	32	16	16	0.003*

* Significant #Fisher's Exact Test; Other P values for categorical data were calculated by Chi-square test with Yates correction

IBS was detected to be associated with other symptoms and medical disorders. Gastrointestinal symptoms in the form of epigastric pain, early satiety, heartburn and vomiting were significantly predominant in the IBS group than others. Some participants had chronic diseases. Four students had hypertension, 4 had hyperthyroidism, 22 had anaemia, 8 had depression, and 22 had anxiety. Thirty-two students were on chronic medications including antacids, proton pump inhibitors, antidepressants, and laxatives. Psychiatric disorders like anxiety and depression were also significantly associated with IBS (P< 0.001, P =0.005) respectively. A high percentage of IBS students were on chronic medical therapy (P =0.03). However, no significant association between IBS and hypertension, hyperthyroidism or anaemia was detected (Table 3). Multivariate logistic regression demonstrated that anxiety, depression, taking caffeine, low fruits and vegetable intake, chronic medications' intake were significant predictors for the development of IBS symptoms (Table 4).

**Table 4:** multivariate analysis of risk factors for irritable bowel syndrome

Risk factor	Odds ratio	95.0% Confidence interval	P – value
Food intolerance	1.295	0.495-3.387	0.598
Medications	3.441	1.288-9.196	0.014*
Caffeine use	2.832	1.168-6.864	0.021*
Milk and milk products	1.659	0.663-4.150	0.279
Food without fruits or vegetables	4.694	1.889-11.669	0.001*
Anxiety	4.243	1.027-17.521	0.046*
Depression	23.267	2.991-180.984	0.003*

## Discussion

The significant heterogeneity in IBS prevalence had been noticed in different areas of the world and different sectors of peoples [19, 20]. In the Arab countries, out of 12 studies, a systematic review reports for IBS prevalence were ranging from 8.9% to 79.7% [21]. Moreover, of IBS prevalence is 9.3-35.3% among medical students worldwide [22].

According to Rome III criteria, IBS was prevailing among the medical students and residences in 27.5% of our study sample. This was in agreement with a study carried out in Al-Neelain University College of Medicine Sudan which revealed that 27.2% of medical students had IBS [23]. Nevertheless, 31.7% of Ain Shams University medical students had IBS [17] In Saudi Arabia; IBS achieved the highest prevalence among medical students. Out of 90 medical students, 38 (42.22%) were affected by the IBS [24]. While in another study in Saudi Arabia in Al Maarefa University, 28.5% of medical students were suffering from IBS [25]. However, the results were different when the studies were carried on all students (medical and nonmedical) in Damascus University, Damascus, Syria [26], and Lebanon [27] as the recorded IBS prevalence was much lower (17% and 20% respectively). This might be due to the longer duration of courses, too many exams, and lots of study materials faced by medical students during their study period.

On the other hand, two studies in Malaysia, one in Melaka-Manipal Medical College [28], and another in Malaysian Private University [29], assessed that 10% and 14.7% of medical students suffered from IBS, respectively. In the previous study of medical students in Canada [30], China [31], Nigeria [32], Korea [16], and Iran [15], the prevalence of IBS were 20.5%, 33.3%, 14.4, 29.2% and 12.6% respectively. In different countries, these variations in prevalence might be due to cultural, dietary, and ethnic patterns. Also, these variations could be related to the sample sizes, age ranges, and diagnostic criteria used throughout various studies [33].

Our study was parallel to Muneer *et al*. who showed no statistically significant association between IBS and gender [34]. However, Elhosseiny *et al*. detected that the majority of IBS cases were females (P =0.006) [17]. Furthermore, studies in Saudi Arabia [24] and Malaysia [29] demonstrated that males reported IBS symptoms more than female medical students. Consistent with several other studies [31, 32, 35]; we found the IBS-mixed subtype (64%) more prevalent among our sample population.

This study showed no significant associations between smoking status, family status, and academic degree on one side and IBS symptoms on another side, which were compatible with the results of Elhosseiny *et al*. [17]. On the contrary, research was carried out in Saudi Arabia showed a strong relation between smoking and IBS among medical students [36]. This difference could be explained that a little number of smokers was in our study sample. Our findings were also in the accordance with Jemilohun *et al*. as we found no significant relationship between age, marital status and IBS prevalence among medical students [32]. Directly opposed to our observations, a study in Saudi Arabia found that sleeping less than 8 hours/day was more associated with development of IBS [33]. The Malaysian survey, however, did not find statistically significant IBS among students with sleep disturbances [29].

Regarding feeding habits, the present study detected a statistically significant relationship between IBS and consumption of some foods and drinks such as coffee, milk products, vegetables and fruits intake. These findings were in line with the study in Saudi Arabia, which found that dietary factors caused 15.5% of IBS symptoms [37]. However, Jung *et al*. Elhosseiny *et al*. and Wani *et al*. failed to find the relationship between IBS and consumption of foods among medical students who were supposed to be less cautious with their diet [16, 17, 24]. Regarding food allergy, our study detected that IBS manifestations were more prevalent in people with food intolerance to special types. Similar findings were reported by Chatila *et al*. who observed; in their study; that IBS patients reported more likely food intolerance [38]. The impact of food allergy is quite well established in activating or intensifying IBS symptoms and is the basis for the low fermentable oligosaccharides, disaccharides, monosaccharides and polyols (FODMAP) diet [39].

In the present analysis, the students with IBS were significantly presented by epigastric pain (P<0.001), early satiety (P=0.032), heartburn (P=0.004), and vomiting (P<0.001). However, Dissanayake *et al*. found diarrhoea to be the most common symptom (40%), followed by vomiting, abdominal distension/bloating (33.6%) then abdominal pain (32.9%) [28]. There was a significant association between anxiety, depression and IBS (P<0.001 and P=0.005 respectively). The results of many other previous studies affirmed our result [17, 29, 32]. Furthermore, Dissanayake *et al*. revealed that medical students with depression were 16.91 times more likely to get IBS [28]. This could be explained by, the immense academic load making medical students under excessive stress [40]. Furthermore, rising attention is being given to the actual impact of psychological distress on IBS pathogenesis, intensity, course, and outcome. IBS symptoms worsen the quality of life of medical students; hence, the associated risk factors should be studied. Curative therapy for IBS is still not known. However, preventive measures against risk factors, raising awareness of the society, and dietary adjustments will be helpful. [41].

There were few limitations, first, our survey was voluntary to participate in, and the response rate was 70%, which led to a small sized sample. Second, the results were assessed through a self-administered questionnaire (Rome III), without performing gastrointestinal endoscopy to confirm the absence of organic colonic disorders. However, these scores have been largely used with good reliability [18]. Third, this survey was restricted to medical students, who are knowledgeable about IBS. So we can't generalize our findings to the general Egyptian populations.

## Conclusion

In conclusion, 27.5% of Egyptian medical students, in this study, met the Rome III criteria for IBS diagnosis. Risk factors for IBS among them included anxiety, depression, taking caffeine, low fruits and vegetables intake, and chronic medications' intake. We recommend; regarding medical students, constructing educational programs in Egyptian medical colleges from the first year. So that the students can be aware of this functional condition and its risk factors they may face as stress and an unbalanced diet. Also, more studies are required in the future to demonstrate the existing prevalence of IBS and its related factors including different levels and cultures of Egyptian populations.

### What is known about this topic

- Irritable bowel syndrome has higher prevalence in medical field students in comparison to general population;
- Psychological factors and sleep disorders are associated with IBS.

### What this study adds

- Medical students in Egypt are in high risk for IBS because stress of study and frequent examinations, bad food habits, and excessive caffeine intake;
- Anxiety and depression are associated with IBS in Egyptian medical students.
